# Roseolovirus-associated encephalitis in immunocompetent and immunocompromised individuals

**DOI:** 10.1007/s13365-016-0473-0

**Published:** 2016-08-18

**Authors:** Joseph Ongrádi, Dharam V. Ablashi, Tetsushi Yoshikawa, Balázs Stercz, Masao Ogata

**Affiliations:** 1 Institute of Medical Microbiology, Semmelweis University, Nagyvárad tér 4, Budapest, 1089 Hungary; 2 HHV-6 Foundation, 1482 East Valley Road, Santa Barbara, CA 93101 USA; 3 Department of Pediatrics, Fujita Health University School of Medicine, 1-98, Kotsukake-cho, Dengakugakolo, Toyoake, Aichi 470-1192 Japan; 4 Department of Medical Oncology and Hematology, Oita University Hospital, Hasama-machi, Yufu City, 879-5593 Japan

**Keywords:** Human herpesvirus 6 and 7, Post-transplant limbic encephalitis, Glial cell destruction, Intrathecal overexpression of proinflammatory cytokines, Monitoring CSF viral load, Empirical ganciclovir and foscarnet therapy

## Abstract

The roseoloviruses, human herpesvirus (HHV)-6A, HHV-6B, and HHV-7, can cause severe encephalitis or encephalopathy. In immunocompetent children, primary HHV-6B infection is occasionally accompanied by diverse clinical forms of encephalitis. Roseolovirus coinfections with heterologous viruses and delayed primary HHV-7 infection in immunocompetent adults result in very severe neurological and generalized symptoms. Recovery from neurological sequelae is slow and sometimes incomplete. In immunocompromised patients with underlying hematological malignancies and transplantation, frequent single or simultaneous reactivation of roseoloviruses elicit severe, lethal organ dysfunctions, including damages in the limbic system, brain stem, and hippocampus. Most cases have been due to HHV-6B with HHV-6A accounting for 2–3%. The most severe manifestation of HHV-6B reactivation is post-transplantation limbic encephalitis. Seizures, cognitive problems, and abnormal EEG are common. Major risk factors for HHV-6B-associated encephalitis include unrelated cord blood cell transplantation and repeated hematopoietic stem cell transplantation. Rare genetic disorders, male gender, certain HLA constellation, and immune tolerance to replicating HHV-6 in persons carrying chromosomally integrated HHV-6 might also predispose an individual to roseolovirus-associated brain damage. At this time, little is known about the risk factors for HHV-7-associated encephalitis. Intrathecal glial cell destruction due to virus replication, overexpression of proinflammatory cytokines, and viral mimicry of chemokines all contribute to brain dysfunction. High virus load in the cerebrospinal fluid, hippocampal astrogliosis, and viral protein expression in HHV-6B-associated cases and multiple microscopic neuronal degeneration in HHV-7-associated cases are typical laboratory findings. Early empirical therapy with ganciclovir or foscarnet might save the life of a patient with roseolovirus-associated encephalitis.

## Introduction

Viral encephalitis is a medical emergency. It is an aseptic inflammatory process of the brain parenchyma associated with clinical evidence of brain dysfunction, significant morbidity, and mortality (Michael and Solomon 2012). It has two forms: epidemic and sporadic. The characteristics of brain involvement and prognosis of the disease depend on the pathogen and the physical state of the host (Kawamura et al. 2013; de Ory et al. 2013; Zerr et al. 2011). Its outcome depends on rapid clinical and microbial diagnosis, and the prompt administration of adequate antiviral therapy (Michael and Solomon 2012). Viral encephalitis frequently involves the meninges, causing meningoencephalitis, or the spinal cord, eliciting encephalomyelitis. Encephalopathy is mediated via metabolic processes and can be caused by systemic infection that spares the brain (Chapenko et al. 2016; Miranda et al. 2011; Raspall-Chaure et al. 2013; Steiner et al. 2010).

Human herpesvirus 6 species A and B (HHV-6A and HHV-6B; Ablashi et al. 1991; Dagna et al. 2013; Salahuddin et al. 2007) and HHV-7 (Frenkel et al. 1990) have been regarded as lymphotropic and neurotropic viruses (Ablashi et al. 2000; Chapenko et al. 2016; Savolainen et al. 2005). In Africa, 86–100% of infants acquire HHV-6A without specific symptoms (Bates et al. 2009; Hall et al. 2006; Kasolo et al. 1997; Sjahril et al. 2009), but a recent study has shown that in biobanked sera specimens from hospitalized Zambian children between 3 weeks and 2 years of age who were admitted with febrile illness, HHV-6B DNAemia was predominant over HHV-6A (20.5 % versus 0.3 %; Tembo et al. 2015). Primary infant infections in Europe, USA, and Japan are predominantly HHV-6B (97–100%) and result in a self-limited fever, with 10–24% of children also developing the associated skin rash, exanthem subitum (roseola infantum; Hall et al. 1994, 2006; Zerr et al. 2005). Both HHV-6A and HHV-6B are implicated as etiological agents or cofactors for a diverse array of pathological conditions in the central nervous system (CNS), such as different clinical forms of encephalitis, encephalopathy, epilepsy, febrile convulsions, meningitis, multiple sclerosis (Alvarez-Lafuente et al. 2007; Crawford et al. 2007; Ongradi et al. 1999c; Yao et al. 2010a), chronic fatigue syndrome (CFS; Ablashi et al. 2000; Komaroff 2006) and tumors (Crawford et al. 2009; Kawabe et al. 2010). All three virus species establish a life-long latency in the immune system and influence the function of immune cells via aberrant cytokine and chemokine patterns or by producing their own chemokines and chemokine receptors (Clark et al. 2013; Crawford et al. 2009; Dagna et al. 2013; Yoshikawa et al. 2011). HHV-6A and HHV-6B persist in the brain (Luppi et al. 1995; Yoshikawa et al. 2000) and can induce neuroinflammation via interleukin (IL) production (Yao et al. 2010b). Single, simultaneous, or consecutive reactivation also elicits debilitating conditions with involving CNS diseases (Holden and Vas 2007; Lautenschläger and Razonable 2012). HHV-6 species are able to transactivate heterologous viruses, especially HIV (Corti et al. 2011; Ongrádi et al. 2011) and human endogenous retroviruses (HERVs; Tai et al. 2009; Turcanova et al. 2009), which cause brain damage. Reactivation of HHV-6 in patients with AIDS can result in encephalitis or meningoencephalitis (Knox et al. 1995). The presence of HHV-6 DNA and proteins in demyelinated brain areas suggests that they play an active role in neurological complications (Knox and Carrigan 1995; Knox et al. 1995; Saito et al. 1995).

HHV-6A and HHV-6B, but not HHV-7, can integrate into the telomeric region of human chromosomes (Arbuckle et al. 2010; Luppi et al. 1995; Montoya et al. 2012; Morissette and Flamand 2010; Lautenschläger and Razonable 2012; Pantry et al. 2013). In approximately 0.2–0.35 % of the population in Japan and Canada (Gravel et al. 2013b; Tanaka-Taya et al. 2004) and 0.8–0.85 % of the population in the USA and UK (Hall et al. 2010; Leong et al. 2007; Ward et al. 2006), HHV-6 is transmitted vertically by standard Mendelian inheritance (Morissette and Flamand 2010). Cells with chromosomally integrated HHV-6 (ciHHV-6) are transmitted to cell and organ transplant recipients with unknown consequences. CiHHV-6 has been shown to activate *in vitro* (Arbuckle et al. 2010) and *in vivo* (Endo et al. 2014), and affected individuals can produce infectious virions from their own integrated strains that are activated under conditions of extreme immunosuppression (Endo et al. 2014). It would therefore be helpful for ciIHHV-6 status to be identified perinatally (Hall et al. 2010). Bans on donations of organs, blood, germ cells, and hematopoietic stem cells from these individuals have been suggested (Flamand 2014; Pellett et al. 2012; Wittekindt et al. 2009). Women with ciHHV-6 can pass their activated HHV-6 to the fetus transplacentally (Hall et al. 2010) and the strains of their congenitally infected fetuses are identical to their integrated strain (Gravel et al. 2013a). CiHHV-6 patients may have tolerance and an inability to control exogenous HHV-6A or B infections (Pantry et al. 2013; Tanaka-Taya et al. 2004), and some of them suffer from neurological symptoms (Lee et al. 2011; Pantry et al. 2013) for several years with remittences and relapses (Montoya et al. 2012). In a recent series of 366 adult allogeneic HSCT recipients, 4 % of HHV-6 positive individuals were ciHHV-6 carriers (Quintela et al. 2016).

HHV-7 is ubiquitous worldwide. By the age of 4 years, approximately 70 % of children have contracted the infection (Adams and Carstens 2012; Ongradi et al. 1999b), but a recent study in Sub-Saharan Africa found that most primary infections appear to occur in young neonates, and subsequently, a very stable prevalence of around 20 % among children >12 months of age was shown by DNAemia. The same study found double infections involving CMV, HHV-6B, or HHV-7 in 3.0–8.9 % of cases, while triple infections involving CMV, HHV-6B, and HHV-7 occured in 2 and 0 % of coinfections involved HHV-6A (Tembo et al. 2015). In children, HHV-7 might elicit exanthem subitum; while in seronegative adults, it might induce pityriasis rosea (Drago et al. 1997; Vág et al. 2004). HHV-7 also invades the brain (Chapenko et al. 2016; Martikainen et al. 2013). Primary infection occasionally induces febrile convulsions or encephalitis and primary infection was found in 8–9 % of children suspected of encephalitis in the UK (Ward 2005). Reactivation of HHV-7 in immunocompromised conditions elicits a wide array of severe CNS diseases. HHV-7 can transactivate HHV-6B and human parvovirus B19 (PV-B19; Ongrádi et al. 2000). Simultaneous HHV-6B, HHV-7, and PV-B19 infection was demonstrated in some patients with myalgic encephalitis (ME/CFS; Chapenko et al*.* 2012a). However, HHV-7 does not activate HIV-1 (Ongrádi et al. 2011) or HERV-K18 (Oakes et al. 2013). HHV-7 has profound immunomodulating activity through altering the cytokine profile and encoding chemokine receptors (Atedzoé et al. 1999).

HHV-6A and HHV-6B ( Ablashi et al. 2014; Adams and Carstens 2012) are discussed separately; the general term HHV-6 is reserved for studies where the distinction was not made (Lautenschläger and Razonable 2012). Roseoloviruses might induce both encephalitis and encephalopathy (Chapenko et al. 2016). These clinical entities are discussed individually when possible. The majority of related publications are case studies or small cohorts up to one or two dozen individuals (Al-Zubeidi et al. 2014; Hill et al. 2012). Unusual clinical presentations and descriptions as well as inconclusive laboratory results are common because of the wide range of biomarkers that have been studied (Fay et al. 2015; Maramattom 2015; Ward et al. 2002; Yamamoto et al. 2014; Zerr et al. 2011). These difficulties hinder unambigous generalization concerning disease course and roseolovirus etiology (Esposito et al. 2015; Hill et al. 2015a, b).

## Clinical presentation of roseolovirus-associated encephalitis

An acute encephalitis might occur either after a primary infection in an immunocompetent individual or after becoming immunocompromised due to an underlying condition. Over the past 10 years, viral encephalitis has gained recognition as an important complication in cell and organ transplantation settings (de Ory et al. 2013; Ibrahim et al. 2005; Imataki and Uemura 2015; Ljungman 2002; Steiner et al. 2010; Michael and Solomon, 2012; Scheurer et al. 2013).

### Encephalitis in immunocompetent individuals

Encephalitis is suspected in small children when an altered level of consciousness, significant change in personality, cognitive dysfunction, or focal neurological symptoms persist for ≥24 h, and other causes are excluded. The disease is accompanied by headache, nausea, temperature of ≥38 °C, and specific laboratory results (Ibrahim et al. 2005). Long before the etiology of ES was established, its association with occasional convulsions and encephalopathy had been observed (Berenberg et al. 1949). Meningoencephalitis associated with HHV-6-related exanthem subitum was reported later (Ishiguro et al. 1990). Since the first published fatal case (Asano et al. 1992), primary HHV-6B infection with or without manifestation of exanthem subitum but accompanied by febrile seizures, acute encephalitis, meningitis, or demyelinating disease in children and adults have been described (de Ory et al. 2013; Yoshikawa et al. 2009). Febrile seizures occur in 20–43 % of children from 6 months to 5 years of age (Epstein et al. 2012; Laina et al. 2010). The first episode is frequently attributed to primary HHV-6B infection, whereas further episodes are not (Yavarian et al. 2014). Eight to 20 % of children with primary HHV-6 infection demonstrate febrile seizures as a main manifestation (Asano et al. 1994). There is a potential link between febrile seizures, temporal lobe epilepsy (TLE), and hippocampal sclerosis (Li et al. 2014). HHV-6B encephalitis has various types of clinical courses, including acute necrotizing encephalopathy (ANE), hemorrhagic shock and encephalopathy syndrome (HSES), and acute encephalopathy with biphasic seizures and late reduced diffusion (AESD; Hoshino et al. 2012; Kawamura et al. 2013, 2014). In 40 % of cases, low HHV-6 load has been detected in the CSF (Yao et al. 2009). Convulsions are frequently atypical and can sometimes lead to *status epilepticus* (Al-Zubeidi et al. 2014; Asano et al. 1992; Iwasaki et al. 2014; Shahani 2014). Of 169 infants with *status epilepticus*, 32 % had active HHV-6B and 7 % had HHV-7 infection. Of those, with active infections, a primary infection was found in 70 % of HHV-6B and 67 % of HHV-7 status epilepticus cases with the rest identified as reactivation (Epstein et al. 2012). The exact fatality rate of HHV-6B-associated encephalitis at the time of primary infection remains unclear. Disabling sequelae (e.g., visual impairment, speech disturbance, persistent hemiplegia, quadriplegia, and mental retardation) are frequent in children after an HHV-6B encephalitis or meningoencephalitis course (Bozzola et al. 2012; Kawamura et al. 2014; Raspall-Chaure et al. 2013). A survey estimated 60 exanthem subitum-associated encephalitis cases with two fatal outcomes annually in Japan and almost half of those children experience severe neurological sequelae (Yoshikawa et al. 2009).

In apparently normal children, reports draw attention to the association between rare genetic disorders and poor neurologic outcome following HHV-6B infection. Two boys 10 and 12 months old, presented with HHV-6B encephalitis, and ultimately died due to Alpers–Huttenlocher syndrome. They were treated with ganciclovir resulted in declining viral load without neurological improvement. Both children were heterozygotes for mutations in the mitochondrial polymerase gamma gene (POLG). This phenotype could be unmasked and/or exacerbated by HHV-6B infection, potentially contributing to clinical deterioration. Mutations in POLG have recently been identified in a number of neurological syndromes including encephalopathy and seizures (Al-Zubeidi et al. 2014). The thermolabile phenotype of another mitochondrial enzyme, carnitine palmitoyl-transferase 2, has also been implicated in acute HHV-6B-associated encephalopathy. This enzyme is found in Japanese people and is involved in the production of ATP. During high fever, the enzyme is inactivated leading to an energy crisis (Kobayashi et al. 2013).

Encephalitis in immunocompetent children due to primary HHV-7 infection is rare. An acute HHV-7 infection in the CSF might be accompanied by severe neurological and generalized symptoms, including encephalitis, meningoencephalitis, encephalopathy, facial palsy, vestibular neuritis, febrile or focal seizures, severe headache, somnolence, fatigue, nausea, vomiting, fever up to 39.8 °C, photosensitivity, lethargy, impaired orientation, difficulties in walking, tendency to fall to one side (straddle gait), tiredness, sudden onset of dizziness, and comatous state (Chan et al. 2002; Ward 2005; Epstein et al. 2012; Pohl-Koppe et al. 2001; Venancio et al. 2014). Delayed primary HHV-7 infection in older children and adolescents can cause more serious neurological complications. In 2,972 hospitalized pediatric patients (mean age 10.1 years), HHV-7 DNA was found in the CSF of 57 concomitant with meningitis, encephalitis, or severe CNS disorders (acute disseminated encephalitis/ADEM/optic neuritis, transverse myelitis, cranial nerve VI palsy, Guillan–Barré syndrome). Learning disabilities persisted even after clinical recovery (Schwartz et al. 2014). In an 11-year-old male with encephalitis symptoms, MRI demonstrated FLAIR abnormalities supratentorially and infratentorially, most prominently involving the brainstem, with diffuse post-contrast enhancement in the pons and a ring-enhancing lesion in the cerebellum subsequently evolving scattered microhemorrhages, necrosis of the pontine lesions. CSF was notable for the presence of HHV-7, leukocytosis (lymphocyte predominance), red cells, glucose, and protein. Sequelae (one-sided weakness, ataxia, and dysarthric speech) gradually normalized (Fay et al. 2015). In adults, primary HHV-7 infection-associated encephalitis is rare but is concomitant with severe generalized symptoms, such as urinary retention, respiratory failure, and flaccid paralysis of the limbs progressing to quadriplegia (Miranda et al. 2011; Schwartz et al. 2014; Ward 2005; Ward et al. 2005). Recently, the combination of a genetic disorder (anti-N-methyl-D-aspartate receptor dysfunction) and HHV-7 DNA positivity in the CSF has been described in a 9-year-old boy with typical neurological symptoms (Venancio et al. 2014).

No encephalitis cases have been published after a simultaneous primary infection with HHV-6 and HHV-7. In children presenting with febrile *status epilepticus*, primary HHV-7 infection and consequent HHV-6B reactivation were found (Epstein et al. 2012). Coincident HHV-7 and cytomegalovirus (CMV) infection-associated encephaloradiculomyelitis has been described (Ginanneschi et al. 2007). In a cohort of 22 cases of herpes simplex virus (HSV)-induced encephalitis, three had both HSV and HHV-6 in their CSF, two of whom died (Ward 2005).

Herpesviruses and other latently carried viruses frequently reactivate in immunocompetent individuals with or without clinical symptoms. In a 64-year-old man, detection of HHV-6 in the CSF was associated with encephalo-myocarditis (Maramattom 2015). Occasionally, double infection by HSV-1 and CMV or HSV-1 and HHV-6 was detected in the CSF of encephalitis patients by polymerase chain reaction (PCR; Ibrahim et al. 2005). Significantly higher detecion frequency of single HHV-6B and concurrent HHV-6B+HHV-7 DNA was found in pia mater meninges, the frontal lobe, and olfactory nerve in individuals with unspecified encephalopathy (UEP) compared to the control group (Chapenko et al. 2016). Additionally, HHV-7 may induce indirect effects in the body which act as a risk factor for CMV reactivation and diseases (Ljungman 2002). In adults with ME/CFS, frequent reactivation of HHV-7, PV-B19, HHV-6B+HHV-7, HHV-7+PV-B19, and HHV-6B+HHV-7+PV-B19 was concomitant with impaired memory or concentration, unrefreshing sleep, new type of headache, and rheumatic symptoms. High viral load in peripheral blood lymphocytes was accompanied by high plasma levels of proinflammatory tumor necrosis factor (TNF)-alpha, IL-6, and undetectable levels of anti-inflammatory IL-4 (Chapenko et al. 2012a). In patients with influenza virus-associated encephalopathy, no association with primary or reactivated HHV-6 or HHV-7 infection was found (Kawada et al. 2003).

### Encephalitis following roseolovirus reactivation in immunocompromised patients

Despite frequent (40–60 % of cases; Deconinck et al. 2013; Zerr et al. 2011) HHV-6 reactivation after stem cell (SCT), bone marrow (BMT), and solid organ (SOT) transplantation, a small percentage of patients develop HHV-6-associated encephalitis (Hill et al. 2012; Ljungman 2002; Ogata et al. 2010; de Oliveira et al. 2015; Sakai et al. 2011; Zerr et al. 2011). Viremia is common 2–6 weeks after transplantation (Yoshikawa et al. 2002b; Zerr 2006; Zerr et al. 2011), but early viral reactivation in the brain might cause encephalitis (Drobyski et al. 1994; Hirabayashi et al. 2013; Ishiyama et al. 2011; Ward 2005). Onset of encephalitis was recorded at a median of 15 (Zerr et al. 2011), 18 (Sakai et al. 2011), or 24 days post-transplantation (Zerr 2006), but it might develop somewhat earlier (Hill et al. 2015a, b) or several weeks later (Drobyski et al. 1994). Patients generally were young—median age, 35 years; range 6–54 years (Zerr 2006) and median 40.5 years elsewhere (Sakai et al. 2011). Occasionally, elder recipients might suffer from HHV-6 encephalitis (Hill et al. 2015a, b) and *status epilepticus* (Shahani 2014). CNS symptoms can be partially different from those seen in primary HHV-6 infection-associated encephalitis and often include depressed consciousness, confusion, disorientation, insomnia, memory problems (especially short-term memory), clinical and electrographic seizures, and imaging abnormalities (Drobyski et al. 1994; Hirabayashi et al. 2013; Singh and Paterson 2000; Zerr 2006; Zerr et al. 2011). Post-transplantation acute limbic encephalitis (HHV-6-PALE) with high mortality is the most typical and severe manifestation, especially after HSCT (Howell et al. 2012; Kawamura et al. 2013, 2012; Ogata et al. 2010; Raspall-Chaure et al. 2013). Small amounts of viral DNA may signify an active infection (Yao et al. 2010a). Sequelae due to hippocampal sclerosis and dysmaturation of the temporal lobe after HHV-6-PALE might develop as epilepsy or loss of attained language and social skills (Howell et al. 2012). Both direct effects of chronic or reactivated HHV-6 infection and autoimmune mechanisms are suspected (Raspall-Chaure et al. 2013). In one case, posterior reversible encephalopathy syndrome (PRES) has been associated with HHV-6B infection (Kawamura et al. 2013, 2012), and in another case, PRES was elicited by combined chemotherapy consisting of drugs known to activate HHV-6, although its presence was not tested (Tsukamoto et al. 2012). Host defense systems that are suppressed due to reasons other than transplantation might also predispose to HHV-6 reactivation and encephalitis. A 2-month-old boy with X-linked severe combined immunodeficiency (X-SCID) and ciHHV-6A was hospitalized with hemophagocytic syndrome and a high HHV-6A load in his blood. After HSCT and antiviral treatment he became asymptomatic. Although he avoided encephalitis or encephalopathy, similar case studies might describe CNS involvement (Endo et al. 2014).

HHV-7 reactivation from neurolatency has been associated with encephalitis in BMT patients (Ljungman 2002). HHV-7 reactivation has not been observed at a constant time post-SCT (Inazawa et al. 2015; Savolainen et al. 2005). After receiving combined immunosuppressive chemotherapy, irradiation, and HSCT for acute lymphoblastic leukemia (ALL), a small girl developed visual and hearing impairment, bulbar dysfunction, confusion, cardiorespiratory insufficency, and died in spite of ganciclovir therapy. Her CSF sample was positive contrary to other leukemic children who did not have encephalitis or HHV-7 in the CSF. This suggests an active role for HHV-7 in CNS damage. These authors estimated that HHV-7 accounts for 1.3–5.7 % of pediatric encephalitis cases (Chan et al. 2002), while others found an incidence of 2 (de Oliveira et al. 2015) or 8 % (Inazawa et al. 2015) after allogeneic HSCT. In addition, HHV-7 reactivation-associated encephalitis has followed initial first course of encephalitis induced by HHV-6 after HSCT. Several weeks of renewed foscarnet therapy resulted in neurological improvement (Holden and Vas 2007). HHV-7 reactivation after transplantation may also lead to CMV reactivation and CMV disease (Chan et al. 1997; Chapenko et al. 2012b; Kidd et al. 2000). HHV-7 viremia was found mostly (94 %) before CMV reactivation, which suggests a possible interaction between these Betaherpesviruses (Tomaszewska et al. 2014). HHV-7 may worsen immunodeficiency through aggravating cytokine dysfunction (Ginanneschi et al. 2007). Its ability to induce high levels of IL-10 might contribute to CNS disorders (Ongrádi et al. 1999a).

## Pathology and pathogenesis

HHV-6A and HHV-6B infect different cells (Donati et al. 2005; Lusso et al. 1991, 1995) and that phenomenon might determine the clinical findings in the CNS (Yao et al. 2010a). Species A gains access to the CNS by crossing the blood-brain barrier (BBB) through the olfactory ensheating cells (OEC). These cells support HHV-6A replication *in vitro* and produce proinflammatory cytokines, such as IL-6, chemokines CCC-1, and CCL5 (regulated upon activation normal T cell expressed and secreted, RANTES). As with other viruses that utilize the olfactory route, the presence of HHV-6 in limbic tissues can lead to limbic encephalitis (Harberts et al. 2011). Recently, HHV-6B has been found in the endotheliocytes and oligodendrocytes of the olfactory tract and the frontal and temporal lobes. High HHV-6B load in the pia mater suggests that HHV-6 may also use this loose vascular coat as a pathway to enter CNS (Chapenko et al. 2016). HHV-6A replicates in neuronal stem cells (De Filippis et al. 2006), progenitor-derived astrocytes (Donati et al. 2005), and oligodendrocyte progenitor cells (Ahlqvist et al. 2005). HHV-6B infection in the astrocytic cell line U251 leads to abortive infection, whereas HHV-6A infection leads to replication (Donati et al. 2005; Yoshikawa et al. 2002a). Infection of oligodendrocytes may be associated with multiple sclerosis, whereas productive infection of astrocytes may be more common in other CNS disorders such as mesial temporal lobe epilepsy (MTLE) and encephalitis (Donati et al. 2003; Drobyski et al. 1994). HHV-6 displays tropism for hippocampal astrocytes (Ogata et al. 2010; Zerr, 2006) and causes neuronal loss in the infected areas (Howell et al. 2012; Yao et al. 2010a) after HSCT (Sakai et al. 2011) or BMT (Drobyski et al. 1994). In hippocampal specimens obtained from patients with TLE, decade-long persistence of HHV-6B was proven (Esposito et al. 2015). HHV-6A productively infects glial cells, while HHV-6B causes their persistent infection (refs in Yao et al. 2010a). In pediatric gliomas that were HHV-6 positive, 72 % were positive for HHV-6A (Crawford et al. 2009). Species specific immune modulation (Dagna et al. 2013; Ongrádi et al. 2006; e.g., differences in chemokine expression) contributes to the predominance of HHV-6B in neuroinflammatory disease (Buckner et al. 2011). In HHV-6B-associated *status epilepticus* and subsequent TLE (Epstein et al. 2012), there is evidence for a role for CCL2-CCR2 signaling (van Gassen et al. 2008) through molecular mimicry. The N-terminal (NT) peptide fragment of CCL2 (formerly known as monocyte chemotactic protein /MCP/-1) is expressed by HHV-6B U83N as the U83B-NT peptide (French et al. 1999) in latently infected cells and in persons carrying ciHHV-6. Upon binding CCR2, it enhances chemotaxis of CCR2-bearing monocytes. In many infections, CCL2 mediates monocyte movement across the BBB and neuroinflammatory diseases (Buckner et al. 2011). HHV-6A U83A-NT is specific for other chemokine receptors and consequently does not elicit chemotaxis.

Cytokine patterns in the CSF were found to be different in encephalitis cases occurring after primary infections and reactivation in transplant recipients (Ichiyama et al. 2009; Kawamura et al. 2011). In small children presenting fever but no sign of CNS involvement at time of HHV-6B seroconversion, elevated serum IFN-gamma, IL-2, IL-4, and MCP-1 levels in the acute phase and a higher IL-5 level in the convalescent period were shown (Yoshikawa et al. 2011). Infants with primary HHV-6 infection-associated encephalitis show higher serum and CSF levels of IL-6. SCT recipients have a tendency to display hypercytokinemia. Among these patients, elevated levels of IL-6 have been detected before HHV-6 reactivation and progression towards encephalopathy (Ogata et al. 2010). A high CSF level of IL-8 relative to serum levels (Kawabe et al. 2010) and elevated levels of IL-10, IFN-gamma, IL-8, tissue regenerating matrix metalloproteinase (MMP)-9, and tissue inhibitor of matrix metalloproteinase (TIMP)-1 were found in encephalitis patients but not in healthy individuals (Ichiyama et al. 2007; Kawabe et al. 2010; Ogata et al. 2010). Variable serum patterns might be a characteristic of different courses of HHV-6B encephalitis; indeed, the cytokine storm in HSES or ANE has stood in contrast to more modest cytokine changes in AESD (Kawamura et al. 2013). In AESD, significantly elevated serum levels of IL-10 and IL-8 were found, while serum IL-1beta and MCP-1 concentrations were lower than in controls. The patients’ levels of IL-10, RANTES, and monokine induced by IFN-gamma (MIG) were significantly higher in serum than in CSF; IL-6, IL-8, and MCP-1, however, were significantly higher in CSF than in the serum. Serum IL-10 was significantly higher in AESD patients with sequelae than in those without sequelae, and therefore, high IL-10 might signify a poor prognosis. Elevation of MCP-1 levels in CSF may be induced by neurons, astrocytes, or microglia and not leaked from systemic circulation. Higher IL-6 and IL-10 levels are frequently measured in several neurological disorders, including encephalitis (Asano et al. 2010; Ichiyama et al. 2009). Elevated levels of these cytokines may play an important role in the pathogenesis of HHV-6B-associated AESD via recruitment of monocytes/macrophages and neutrophils or via activation of glial cells (Kawamura et al. 2013). *In vitro* reactivation of HHV-6B infection in an astrocytoma cell line resulted in the overproduction of IL-6 and IL-1beta (Yoshikawa et al. 2002a). High CSF levels of IL-1beta and IL-8 triggers vasogenic edema in the brain (Kawamura et al. 2013, 2012). High plasma IL-6 levels were also found in a patient with ciHHV-6 (Montoya et al. 2012). Elevated levels of IL-6, soluble TNF receptor (sTNFR)-1, and CSF IL-1 predict neurological sequelae (Ichiyama et al. 2009; Montoya et al. 2012). These alterations are similar to those found in acute encephalitis induced by other viruses and suggest the existence of a pathway that is partially conserved across species (Hoshino et al. 2012; Kawada et al. 2003; Singh and Paterson 2000).

In male adults with drug-resistant epilepsy, HHV-7 proteins were detected in the astrocytes and oligodendrocytes of hippocampal sclerosis samples. Glial scar, arachnoid cyst, focal cortical dysplasia, and vascular malformation are associated with HHV-7 infection. Beside normal TNF-alpha, IL-1, and IL-6 levels, tumor growth factor (TGF)-beta upregulation found in degenerating neurons of the pyramidal cell layer suggests a possible association with persistent glial-tropic HHV-7 infection and chronic neuroinflammatory conditions via glio-neuronal communication (Li et al. 2014). Simultaneous detection of HHV-6+HHV-7 DNA in the frontal lobe, but not in the temporal lobe of patients with UEP, suggests that their potential interaction can affect the brain through cytokine mediated immunomodulation (Chapenko et al. 2016).

Research on pathomechanism has long been hindered due to a lack of appropriate animal models (Reynaud and Horvat 2013). Upon HHV-6A and HHV-6B infection of murine primary oligodendrocyte precursors, transcription of viral genes was detected, but viral replication did not occur (Mock et al. 2006). Several monkey species carry detectable antibodies due to natural infection. An HHV-6B homolog (PanHV-6) was isolated from chimpanzees (*Pan triglodytes*); however, infection remains asymptomatic (Lacoste et al. 2005). In pig-tailed macaques (*Macaca nemestrina*), experimental HHV-6A infection accelerated simian immunodeficiency virus (SIV) infection and the progression of pathological conditions, but no neurological symptoms have been described (Lusso et al. 2007). Intravenous HHV-6A infection in common marmosets (*Callithrix jacchus*) resulted in neurological symptoms including motor weakness and sensory abnormalities, while infection with HHV-6B remained asymptomatic. HHV-6A and HHV-6B DNA could be occasionally detected in the brain of animals and MRI revealed hyperintense lesions in the brain of one animal. Intranasal HHV-6A infection rarely induced seroconversion or manifested in disease. Neurological symptoms observed with intravenous injection might be due to the immune response raised against HHV-6 rather than to the direct effect of HHV-6 (Leibovitch et al. 2013). Mice with SCID and humanized mice show promise as animal models for learning about HHV-6 pathogenesis (Tanner et al. 2013). In CD46 transgenic animals, HHV-6A, but not HHV-6B, established long-term (9 months) persistence in the brain. Neuroinflammation, such as that associated with chemokine CCL5 (RANTES) production by glial cells via HHV-6A binding to toll-like receptor (TLR)-9, has been documented (Reynaud et al. 2014). Recently, novel Betaherpesvirus DNA related to HHV-7 have been found in the peripheral blood mononuclear cells (PBMC) of African great apes by PCR (Lavergne et al. 2014). On the whole, the animal experiments seem to reflect the findings in human cases.

## Epidemiology and risk factors

### Risk factors for encephalitis associated with primary infection

Roseoloviruses are ubiquitous; the etiological spectrum is not influenced by geography. Most primary HHV-6B infection occurs in the second 6 months of life, while HHV-7 primary infection occurs in the second or third year (Ongrádi et al. 1999b; Ward 2005). This temporal sequence does not apply in children with encephalitis or seizures in which the median age is 1 year for both. Absence of previous HHV-6 infection predisposes to HHV-7-induced neurological morbidity due to lack of cross-reacting antibodies (Ward 2005). The majority of children and adults suffering from HHV-6 and HHV-7 encephalitis are male (Ginanneschi et al. 2007; Kawada et al. 2003; Ongrádi et al. 2013; Pohl-Koppe et al. 2001; Schwartz et al. 2014; Ward et al. 2002), and the highest incidence of encephalitis (70%) occurred in winter (Yavarian et al. 2014). Higher frequency of encephalitis cases in Japan compared to the rest of the world might reflect genetic susceptibility (Kobayashi et al. 2013) or more conscientious monitoring (Yoshikawa et al. 2009). Rare genetic alterationss might predispose to neurological disorders among them HHV-6-associated encephalopathy (Al-Zubeidi et al. 2014). There are also risk factors associated with medical practice. For small children with exanthem subitum, physicians might prescribe unnecessary antibiotics which can have adverse effects in the presence of HHV-6 infection. Amoxicillin (Mardivirin et al. 2010), carbamazepine, and sodium valproate (Mardivirin et al. 2009) have been shown to activate HHV-6 *in vitro*; thus, they might facilitate the development of encephalopathy. Hydrocortisone is a strong enhancer of HHV-6 replication (Arbuckle et al. 2010), and prednisolone derivates given to suppress skin rash might contribute to higher CSF viral load (Hill et al. 2015a, b), but in children with CNS diseases, prednisolone therapy (Kawamura et al. 2013; Pohl-Koppe et al. 2001) helps protect the BBB (Kawamura et al. 2013; Steiner et al. 2010; Venancio et al. 2014; Yamamoto et al. 2015).

### Risk factors for encephalitis associated with virus reactivation

Today, reactivation of roseoloviruses is almost exclusively detected in patients with special conditions. Although HHV-6 reactivation is relatively common among immunocompromised individuals, the incidence of encephalitis among them is low (Drobyski et al. 1994; Ogata et al. 2010; Sakai et al. 2011; Zerr 2006; Zerr et al. 2011). Reactivation may not be a problematic event *per se*, suggesting that certain attributes of the patients may be related to encephalitis or encephalopathy (Yamamoto et al. 2014). Most cases have been due to HHV-6B with HHV-6A accounting for only 2–3 (Sakai et al. 2011) or 11 % (Zerr 2006). In autopsy samples from patients with UEP, only HHV-6B was detected (Chapenko et al. 2016). Certain normal genetic constellations in the immune system might predispose an individual to HHV-6-associated encephalopathy. In a cohort of 130 recipients with acute leukemias and allo-HSCT HHV-6, HLA class I phenotype of recipients was not related to HHV-6 reactivation (Ʃ = 38.1 %), while encephalopathy was more frequent (Ʃ = 5.1 %) in patients with HLA-B* 40.06. This allele might be related to lesser cytotoxic T cell recognition of virus-infected cells (Yamamoto et al. 2014). Younger patients, especially children or adults in their 20s or 30s after HSCT or SCT (Hirabayashi et al. 2013; Singh and Paterson 2000; Zerr 2006; Zerr et al. 2011), are at high risk, as well. Underlying hematologic malignancies with more advanced conditions, allogeneic transplants, unrelated, mismatched or gender mismatched donors, repeated HSCT, and more importantly unrelated umbilical cord blood cell transplantation (UCBT) have been identified as major risk factors (de Oliveira et al. 2015; Hirabayashi et al. 2013; Ishiyama et al. 2011; Ogata et al. 2013b; Quintela et al., 2016; Raspall-Chaure et al. 2013; Sakai et al. 2011; Shimazu et al. 2013; Zerr et al. 2011). In a cohort of HHV-6-PALE patients, 9.9 % developed limbic encephalitis after UCTB and 0.7 % developed the condition after adult-donor HSCT. Death from HHV-6-PALE occurred in 50 % of affected patients undergoing UCBT, while no recipients of adult-donor cells died (Hill et al. 2012). Prevalence of HHV-6 reactivation and encephalitis was also significantly higher in other patient groups receiving UCBT than in patients receiving another stem cell source (72.0 % versus 37.4 % and 8.3 % versus 0.5 %, respectively; Scheurer et al. 2013). UCBT predisposes to platelet engrafment failure (de Oliveira et al. 2015), and along with steroid treatment and anti-thymocyte globulin use, has been confirmed to be a risk factors for viral reactivation after allogeneic HSCT (Inazawa et al. 2015). Similarly, the risk of HHV-7 transmission and reactivation in UCBT recipients is increased. A survey of UCBT donors showed that the mean viral load in latent cases was 1.31 × 10^1^ copies/ml and 1.94 × 10^4^ in active infections (Abedi et al. 2015).

Most recipient children are HHV-6 seropositive prior to SCT. Higher levels post-transplant of either HHV-6 or HHV-7 antibodies, antigenemia, and DNA detection are more frequent in the allogeneic than in autologous recipients. Patients who have undergone total body irradiation (TBI) have faced reactivation of both HHV-6 and HHV-7 more often than those not irradiated (Imataki and Uemura 2015; Savolainen et al. 2005). Males are more frequently affected (Sakai et al. 2011; Hirabayashi et al. 2013; Endo et al. 2014; Ongrádi et al. 2014). For patients with active HHV-6 infections, a slower CD8+ T cells recovery was observed until 6-months post-transplantation, and the overall probability of survival after allogeneic HSCT was diminished (Quintela et al. 2016). HHV-6 has been linked to acute hemorrhagic encephalitis (AHE) in adults; ANE is more frequent in childhood, although HHV-7-induced AHE has been seen recently (Fay et al. 2015).

Roseolovirus-associated encephalitis is rare after SOT, but recipients of heart, lung (Lautenschläger and Razonable 2012), or liver transplant (Dockrell et al. 1999; Singh and Paterson 2000; Yoshikawa et al. 2002b) are at a slightly increased risk. An association has been documented between a high HHV-6 level in the blood and PBMC, and CNS dysfunction and encephalitis (Ogata et al. 2006; Zerr et al. 2011). HHV-6 was detected more frequently in the CSF of transplant recipients with encephalitis than in those without encephalitis (Zerr 2006). Warning signs for onset of encephalitis or encephalopathy are hypercytokinemia, particularly high IL-6 levels in the early phase of SCT because of engraftment syndrome, and graft-versus-host disease (GVHD; Ogata et al. 2010). In allogeneic HSCT recipients, active HHV-6 infection was significatly more often associated with CMV and or BK virus but not EBV co-reactivations (Quintela et al. 2016). Risk factors and clinical outcomes after HSCT differ between single reactivations of HHV-6, CMV, or EBV (Drobyski et al. 1994; Jaskula et al. 2010).

Patients of any age with malignancies (Martikainen et al. 2013) and those who receive HSCT undergo combined chemotherapy regimens, biological therapy (e.g., anti-T cell antibodies), and intracranial or total body irradiation (Drobyski et al. 1994; Imataki and Uemura 2015; Kawamura et al. 2013, 2012; Ogata et al. 2010; Raspall-Chaure et al. 2013; Wittekindt et al. 2009; Zerr et al. 2011). These combinations frequently contain drugs, such as anti-cancer agent histone deacetylase (HDAC) inhibitors, that are able to activate HHV-6 and HHV-7 or other viruses *in vitro* and *in vivo*, particularly in the CNS (Yao et al. 2008). Certain common pharmaceuticals, among them aliphatic acid derivates, anticonvulsant, analgesic, antipyretic, antiemetic, anesthetic or immunosuppressant drugs, and antibiotics can reactivate or enhance an already existing HHV-6 infection *in vitro*. Similar *in vivo* associations have mainly been found in the context of drug-induced hypersensitivity syndrome (DIHS) or drug rash with eosinophilia and systemic symptoms (DRESS). HDAC inhibitor trichostatin A, hydrocortisone, and 12-O-tetradecanoyl-13-acetate (TPA) have all been shown to reactivate ciHHV-6 *in vitro* (Arbuckle et al. 2010; Iwasaki et al. 2014; Mardivirin et al. 2009, 2010; references in Pellett et al. 2012) In an elderly man, glycocorticoid treatment elicited HHV-6 encephalitis (Yajima et al. 2014), and anti-rheumatic therapy in a patient suffering from HHV-7+CMV-associated encephaloradiculomyelitis contained dexamethamethasone (Ginanneschi et al. 2007). Immunosuppression is vital for transplant recipients, and although it may be difficult to alter treatment protocols, the use of these drugs should be carefully considered in the context of an HHV-6 infection, restricted to a minimum, and potential effects on the viruses should be monitored (Arbuckle et al. 2010; Montoya et al. 2012; Pellett et al. 2012).

## Diagnosis

Infection of the CNS is difficult to diagnose. There is no consensus for testing a standard set of biomarkers in suspected roseolovirus-associated encephalitis (Agut 2011; Gautheret-Dejean et al. 2013).

### Clinical manifestations and other relevant information

The patient’s history is mandatory in the assessment of suspected encephalitis. Immunocompromised conditions and use of recreational drugs or medications known to affect the immune system or reactivate latent viruses have to be declared (Steiner et al. 2010). Neurological complications wrongly attributed to routine childhood vaccination may in fact be due to coincident primary virus infection (Ward 2005; Yavarian et al. 2014). Exanthem subitum, pityriasis rosea, skin rash, and fever with concomitant neurological symptoms suggest an association between viruses and CNS disorders (Savolainen et al. 2005; Yao et al. 2010a). Additionally, behavioral, cognitive, visual, and verbal memory functions including amnesia, delirium and disorientation, focal neurological signs, seizures, and autonomous and hypothalamic disturbances reflect disruptions of brain functions (Steiner et al. 2010; Zerr et al. 2011). Other common symptoms reported are hyponatremia, hemophagocytosis, nystagmus, and dysesthesia. Differentiation between HHV-6 encephalitis and Wernicke encephalopathy occurring in pediatric HSCT recipients improves outcome (Sadighi et al. 2015). In cancer patients, neurologic symptoms usually attributable to brain metastases should be evaluated to exclude a non-oncological cause (Mordenti et al. 2013).

### Laboratory procedures

#### Electroencephalography and neuroimaging

EEG and baseline quantitative EEG (QEEG) can show abnormalities prior to neuroimaging. Several abnormalities, especially in the temporal lobe, have been described in both acute or reactivated HHV-6 and ciHHV-6-associated encephalitis, including slow wave activity in primary HHV-6B or HHV-7 infection-associated encephalitis consistent with brain stem involvement. EEG normalization parallels antiviral treatment and clinical improvement (Al-Zubeidi et al. 2014; Iwasaki et al. 2014; Montoya et al. 2012; Pohl-Koppe et al. 2001; Venancio et al. 2014; Ward et al. 2002; Wittekindt et al. 2009; Yamamoto et al. 2015).

Characteristic neuroradiographic signs of HHV-6-associated encephalitis or encephalopathy in both immunocompetent and immunocompromised patients have been recognized using CT scanning (Al-Zubeidi et al. 2014; Kadwabe et al. 2010; Yao et al. 2010a; Zerr, 2006), MRI (Al-Zubeidi et al. 2014; Chapenko et al. 2016; Crawford et al. 2009; Drobyski et al. 1994; Hirabayashi et al. 2013; Kawamura et al. 2013), and FLAIR MRI (Kawamura et al. 2013, 2012; Maramattom 2015; Venancio et al. 2014; Yajima et al. 2014; Yamamoto et al. 2015). Findings include diffuse high intensity of the limbic system around bilateral, temporal, occipital, and frontal areas (Imataki and Uemura 2015). Susceptibility weighted imaging (Iwasaki et al. 2014) and acute phase 2-deoxy-(F-18)-fluoro-D-glucose positron emission tomography (FDG-PET; Hubele et al. 2012) are also used. Signs develop gradually, but they are somewhat delayed as compared to the clinical symptoms (Imataki and Uemura 2015). In AESD, MRI shows no abnormalities during the first 2 days of the illness, then reduced diffusion in the subcortical white matter is generally observed at the time of the second episode of seizures around the exanthematous period. These typical findings are subsequently resolved and brain atrophy is observed in the convalescent period (Kawamura et al. 2014). HHV-6 encephalitis often involves the cortex, hippocampal, and extrahippocampal structures involving the amygdala, entorhinal cortex, thalamus, hypothalamus and deep forebrain structures, cerebellum, and brain stem. Edema, necrotization, and sclerosis are frequently found ( Al-Zubeidi et al. 2014; Corti et al. 2011; Drobyski et al. 1994; Kawamura et al. 2013; Raspall-Chaure et al. 2013; Singh and Paterson 2000; Yao et al. 2010a). In HHV-7 encephalomyelitis (Pohl-Koppe et al. 2001; Ward et al. 2002) and simultaneous HHV-6+HHV-7 (Holden and Vas 2007) reactivation-associated encephalitis, CT and MRI have detected abnormalities in the cortex, cerebellum, and hippocampus. Improvement parallels antiviral therapy, but sequelae have detectable signs. In many cases of encephalitis after primary HHV-7 infection, normal neuroimaging was shown initially, but without proper treatment, progressive brain atrophy could be detected (Schwartz et al. 2014).

#### General studies on the blood and CSF

In HHV-6 encephalitis following HSCT, the CSF is usually normal, except for elevated protein levels that occurs in some but not all cases. Pleocytosis is rarely found (Corti et al. 2011; de Ory et al. 2013; Iwasaki et al. 2014; Kawamura et al. 2013, 2012, 2014; Maramattom 2015; Wittekindt et al. 2009; Zerr 2006). Severe hyponatremia could be prodromal or concomitant with HHV-6-PALE (Kawaguchi et al. 2013; Raspall-Chaure et al. 2013). In HHV-7 encephalitis, laboratory results are extremely disparate. CSF examination might show normal or elevated protein and glucose levels (Chan et al. 2002; Imataki and Uemura 2015; Pohl-Koppe et al. 2001; Ward et al. 2002), pleocytosis, predominantly lymphocytosis (Fay et al. 2015; Schwartz et al. 2014). Coincident HHV-7 and CMV reactivation has resulted in xanthochromia, pleiocytosis with lymphocyte predominance, red blood cells (RBC), high protein level, and oligoclonal IgG bands in CSF (Ginanneschi et al. 2007). Measurements of cytokine and chemokine panels in CSF and serum are progression markers (Kawabe et al. 2010; Kawamura et al. 2014; Ogata et al. 2010; Yoshikawa et al. 2011).

### Microbial diagnostic investigations

#### Roseolovirus specific tests

Identifying clinically relevant HHV-6 or HHV-7 CNS infection is challenging due to their ubiquitous and persistent nature (Agut 2011). Testing should distinguish between HHV-6A and HHV-6B species in all cases (Hill et al. 2015a, b). Detection of roseolovirus harbouring cells in CSF does not prove etiology (Martikainen et al. 2013), but detection of replicating HHV-6 or HHV-7 in the brain tissues would suggest it (Yao et al. 2010a). Brain biopsy is not used in clinical practice, but at autopsy, brain material should be obtained (Chapenko et al. 2016). CSF and brain tissue samples rarely contain infectious virus (Steiner et al. 2010). Cultivation has now been replaced by the detection of specific nucleic acids (NA) by PCR-based technologies on CSF, CSF cells, brain biopsy, tissues, and blood PBMC. The demonstration of viral DNA in blood or saliva alone must not be considered confirmatory in the diagnosis of encephalitis (de Ory et al. 2013; Lautenschläger and Razonable 2012; Pellett et al. 2012; Raspall-Chaure et al. 2013; Zerr 2006). The highest viral yield in CSF is generally obtained when the virus appears transiently during the first week following onset of symptoms (de Ory et al. 2013; Maramattom 2015; Ward 2005) and becomes almost undetectable as the antibody response commences in week three and beyond (Ward 2005). Others have found that the detectability of viruses in the blood increased after HSCT and peaked at 21 days post-transplantation (Inazawa et al. 2015).

The presence of ciHHV-6 must also be considered in encephalitis (Gautheret-Dejean et al. 2013; Zerr 2006; Zerr et al. 2011). CiHHV-6 can be confirmed in acutely ill patients by performing PCR on hair follicle or fingernail samples where active HHV-6 replication is not present (Endo et al. 2014; Hall et al. 2010; Montoya et al. 2012; Ward et al. 2006), although their plasma, serum, and blood always tests positive for HHV-6 (Deconinck et al. 2013; Hall et al. 2010; Pellett et al. 2012; Ward et al. 2006). Individuals with ciHHV-6 will invariably show a high viral load in whole blood (>log 5.5; Pellett et al. 2012; Zerr et al. 2011). CSF HHV-6 levels will depend on the level of cells, and can vary from high levels (10^4^–10^7^ copies/ml; Gautheret-Dejean et al. 2013) to low levels in ciHHV-6 patients with subacute illness (Montoya et al. 2012). Serum and plasma levels vary significantly, depending on the time between phlebotomy and centrifuge, and are of no value in diagnosing or tracking ciHHV-6 patients (Pellett et al. 2012). Fluorescence *in situ* hybridization (FISH) of the patients’ cells can locate ciHHV-6 at chromosomes (Endo et al. 2014). It might occur that one of the HHV-6 species is integrated and either a primary infection or reactivation of the other HHV-6 species or HHV-7 elicits encephalitis. Active infection in ciHHV-6 patients may improve with antivirals (Endo et al. 2014; Kobayashi et al. 2011; Troy et al. 2008; Wittekindt et al. 2009).

Nested PCR (nPCR) with higher sensitivity is commonly used to detect roseoloviruses in CSF and brain materials (Chan et al. 2002; Chapenko et al. 2016; Kawada et al. 2003; Li et al. 2014; Michael and Solomon 2012; Pohl-Koppe et al. 2001; Yoshikawa et al. 2000). Multiplex PCR (mPCR) assays simultaneously detect all herpesviruses in a very small volume of CSF (Agut 2011; de Ory et al. 2013). PCR-based methods are able to distinguish between HHV-6A, HHV-6B, and HHV-7 (Chapenko et al. 2016; Hall et al. 2010; Inazawa et al. 2015; Lautenschläger and Razonable 2012; Tembo et al. 2015; Ward 2005; Zerr et al. 2011). Qualitative mPCR for the most important encephalitis-inducing microbes is employed as a first-line screening test and subsequently real-time PCR is used for quantitative evaluation of the actual microbe (Chapenko et al. 2016; de Oliveira et al. 2015; Inazawa et al. 2015). The finding of HHV-6 DNA in acellular samples is considered a sign of virus replication, but in whole blood and PBMCs and in ciHHV-6 patients an active infection is best proven by quantitating viral mRNA using reverse transcriptase (RT) PCR (Agut 2011; Epstein et al. 2012; Miranda et al. 2011; Ward 2005). Viral load measurements through quantitative PCR (qPCR) before, during, and after onset of encephalitis allow for a more comprehensive understanding of the viral contribution to disease (Agut 2011; Deconinck et al. 2013; Hill et al., 2015a, b; Laina et al. 2010; Ljungman 2002). Low-copy number (around 10^3^/ml) of HHV-6 DNA in CSF have been detected in many of the patients with HHV-6 encephalitis/encephalopathy during primary infection (Kawamura et al. 2011, 2014), whereas CSF samples obtained from post-transplant HHV-6 encephalitis patients have contained significantly higher-copy number (Kawamura et al. 2011). Real-time PCR can establish viral load in a shorter amount of time (Kawada et al. 2003; Yavarian et al. 2014) and constitutes a reliable diagnostic tool enabling timely initiation of appropriate therapy and rapid assessment of the efficacy of antiviral treatment strategies (Imataki and Uemura 2015; Tomaszewska et al. 2014). The minimum detection level of most real-time PCR is low, e.g., two copies per reaction, while the threshold level for the development of HHV-6 encephalitis seems to be around 10^4^ copies/ml plasma (Kawabe et al. 2010; Ogata et al. 2013b). Unfortunately, the absence of international standards for qPCR testing limits our ability to translate these findings between laboratories. High viral loads, up to 2 × 10^6^/ml, are shown throughout the brain, whereas high levels of HHV-6 DNA in peripheral blood could rather be associated with encephalopathy (Hirabayashi et al. 2013; Yamamoto et al. 2014). Encephalitis and myelitis are associated with alteration of the BBB; the detection of viral DNA in CSF by PCR might not discriminate between a CNS and a systemic infection *per se* (Ginanneschi et al. 2007). Comparison of real-time PCR of HHV-7 in the buffy coat samples for latent infection (3.2 %) and plasma samples for active infection (0.4 %) in 825 UCBT donors real-time PCR for HHV-7 was used to test buffy coat samples for latent infection (3.2 %) and plasma samples for active infection (0.4 %) in 825 UCBT donors. These results suggests that this is a useful test to identify donors with active infections so they can be prevented from donating for some time (Abedi et al. 2015).

In autopsy specimens, histopathology has detected increased brain microglia, perivascular lymphocyte cuffing, and scattered focal hippocampal neuronal dropout, which was nonpsecific, but consistent with viral encephalitis (Hill et al. 2015a, b). Roseolovirus antigens can be demonstrated in specific brain cells by immunofluorescent (IF) or immunoperoxidase methods (Li et al. 2014; Savolainen et al. 2005), but these techniques seem unsatisfactory in CSF samples (Ward 2005). In encephalitis cases, high HHV-6 loads in the hippocampus, basal ganglia, insular cortex, temporal lobe, and cingular gyrus have been found. Additionally, astrogliosis and neuronal loss in regions of the hippocampus paralleled HHV-6 protein expression suggesting a productive infection (Al-Zubeidi et al. 2014; Drobyski et al. 1994; Raspall-Chaure et al. 2013; Yao et al. 2010a). In fatal HHV-6 encephalitis, absence of viral inclusions has been reported (Al-Zubeidi et al. 2014; Drobyski et al. 1994). The lack of a significantly greater prevalence of HHV-6B in the hippocampus of patients with UEP than in controls can be an important distinguishing factor between encephalitis and encephalopathy, as a significatly higher prevalence has been found in patients with encephalitis (Chapenko et al. 2016). In HHV-7 encephalitis, multiple foci of microscopic neuronal degeneration, hemorrhage, necrosis, and perivascular lymphocytic infiltration in the brain stem have been shown (Chan et al. 2002; Fay et al. 2015). Using confocal laser-scanning microscopy for TLE biopsies opened the door to simultaneously detecting roseolovirus antigens along with altered cellular components (Esposito et al. 2015).

Intrathecal antibody detection in the CSF of HHV-6 or HHV-7 is strong evidence for etiology. Systemic serological responses should not be considered for diagnosis. In a young child, viral DNA is not always detectable (Epstein et al. 2012), therefore, plasma DNA testing and serological analysis (with fourfold increase in IgG titer or the presence of IgM antibodies) are important in confirming a diagnosis. Combining CSF PCR with serology is important to prove primary HHV-7 infection when investigating CNS disease (Schwartz et al. 2014). Methods must distinguish primary antibody response and pre-existing antibodies from reactivation, and primary infection may be verified by the use of an IgG avidity test. This test is required in immunocompromised patients as antibody titers may have decreased to the point of seronegativity and the secondary antibody response may appear to be that of a primary antibody response. An HHV-6A and HHV-6B specific immunoblotting assay has been developed (Higashimoto et al. 2012), and HHV-7 antibodies can be shown by indirect IF, immunoblot, and immunoassays (Ward 2005).

#### Differential diagnosis

In sporadic encephalitis, clinical symptoms and laboratory results can be very diverse and unusual. Therefore, it is necessary to test for a variety of pathogens, including different herpesviruses, enteroviruses, influenzaviruses, parainfluenzaviruses, adenoviruses, as well as chlamydophila, mycoplasma, toxoplasma, and campylobacter species (de Oliveira et al. 2015; Esposito et al., 2015; Fay et al. 2015; Hill et al., 2015a, b; Inazawa et al. 2015; Maramattom 2015; Martikainen et al. 2013; Quintela et al., 2016; Schwartz et al. 2014; Venancio et al. 2014; Ward et al. 2002; Yao et al. 2008, 2010b). Viral cultures and PCR are commonly performed on CSF and brain tissue, as well as throat and stool specimens (Chan et al. 2002; Holden and Vas 2007). Multivirus real-time PCR (Yamamoto et al. 2015) and DNA microarrays are increasingly used to identify several microbes and their genotypes simultaneously (Boriskin et al. 2004; Miranda et al. 2011; Steiner et al. 2010). Along with HHV-6-associated encephalitis, concomitant acyclovir resistant HSV-1 and adenovirus esophagitis, rotavirus gastroenteritis, and respiratory syncytial virus penumonia were all identified in a child (Ernst et al. 2012). Antigen detection of adenoviruses, HSV, VZV, parainfluenzaviruses (Steiner et al. 2010), and influenzaviruses from throat samples may provide a possible etiology for encephalitis, but these methods are not helpful in diagnosis using CSF samples (Kawada et al. 2003; Steiner et al. 2010). Serum antibody tests can be helpful in retrospective studies, if there is a fourfold rise in antibody titer, but do not help for intial acute case diagnosis. Microbiological tests are required occasionally for measles, mumps, and rubella in countries without effective vaccination programs, as well as arboviruses and zoonosis in epidemic areas (de Ory et al. 2013; Steiner et al. 2010). The simultaneous presence of several antibodies in the CSF suggests BBB damage. Different viruses induce different histological types of encephalitis. The major targets of roseoloviruses are the brain stem (midbrain, pons, and medulla oblongata), limbic area (amygdala and hippocampus), and the medial temporal lobe. Anatomical injuries corrrespond to clinical symptoms. Damage to the brain stem affects consciousness and results in cardiorespiratory failure, while damage to the limbic area results in elevated body temperature, diuretic hormonal abnormalities, ataxia and cognitive and memory impairments (hippocampus and amygdala), and damage to the temporal lobe contributes to speech difficulties. HSV, VZV, CMV, and HIV can induce panencephalitis, and RNA viruses elicit patchy-nodular types. ADEM might follow febrile illnesses or immunization (Hoshino et al. 2012; Steiner et al. 2010). In pediatric patients, tuberculous meningitis (Steiner et al. 2010) and HSV-1, HSV-2, VZV, and CMV infection (Ibrahim et al. 2005) must be excluded. In Japan, influenza virus was shown to be the most common pathogen in cases of encephalopathy, followed by HHV-6 and rotavirus (Hoshino et al. 2012). In another study, HSV-1, VZV, and enteroviruses were found as the most common etiological agents (de Ory et al. 2013). ANE usually following influenzavirus A or enterovirus infections tends to cause bilateral, sometimes hemorrhagic, injury to the thalami. Its familial form is associated with mutations in the nuclear pore protein RANBP2 (refs in Fay et al. 2015). The risk for HAdV-associated encephalitis has been recognised in children with unrelated or cord blood HSCT, GVHD grades III–IV, and lymphopenia. In contrast to HHV-6 and 7, strict isolation is necessary to prevent horizontal transmission from fecal or urine contamination (Matthes-Martin et al. 2012; Mynarek et al. 2013). These data show the importance of differential diagnosis in handling patients and protecting the health care staff from acquiring nosocomial infection. Occasionally, bacterial superinfection (Ljungman 2002) or infection of other organs associated with underlying diseases have to be recognised and treated (Holden and Vas, 2007; Venancio et al., 2014). Death of the patient could be due to an intercurrent infecton (Imataki and Uemura 2015). Clearly, there is a need for further studies on the etiological role and interaction of unrelated neurotropic viruses especially in cases with atypical clinical manifestations (Chapenko et al. 2012a).

### Antiviral therapy, prevention, and prognosis

Primary infection by roseoloviruses usually does not require specific treament. It is not known whether antiviral treatment of primary HHV-6B or HHV-7-associated febrile *status epilepticus* would be beneficial (Epstein et al. 2012). Clinical diagnosis of viral encephalitis before HHV-6 or HHV-7 identification is usually suspected to be of HSV or VZV origin. Acyclovir therapy is administered, but it has a very limited effect or no effect at all against roseoloviruses (Chan et al. 2002; Corti et al. 2011; Fay et al. 2015; Ljungman 2002; Steiner et al. 2010; Tomaszewska et al. 2014; Venancio et al. 2014; Yamamoto et al. 2015; Zerr et al. 2011). When reactivation of HHV-6 or HHV-7 is recognized, anti-roseolovirus medication has to be initiated. Foscarnet and ganciclovir (valganciclovir) are the first line of defense, and cidofovir is the second (Deconinck et al. 2013; Mordenti et al. 2013; Prichard and Whitley 2014; Venancio et al. 2014). In simultanous infections of HHV-6 and CMV or HHV-7 and CMV, ganciclovir was found to be an effective treatment, but it has been determined in another case that the drug showed efficacy against CMV yet was unable to eliminate HHV-7 (Tomaszewska et al. 2014). Recent treatment regimens recommended by different authors are very similar. High-dose ganciclovir (18 mg/kg/day) or 60 mg/kg foscarnet twice a day was empoyed for the treatment of a CNS HHV-6 infection in an immunocompromised host (Imataki and Uemura 2015). Generally, foscarnet 60 mg/kg i.v. every 8 h or 90 mg/kg every 12 h (180 mg/kg/day), or ganciclovir 5 mg/kg i.v. every 12 h are recommended for the treatment of HHV-6 encephalitis after allogeneic stem cell trasnplantation (Zerr 2015). Successful treatment protocol of several HSCT recipients and other patients has been published, but randomized placebo-controlled trials are lacking (Holden and Vas 2007; Ljungman 2002; Pöhlmann et al. 2007; Sadighi et al. 2015). Antiviral therapy might last for weeks to months and the general scheme follows that used in case of CMV reactivation (Deconinck et al. 2013). Clinical symptoms may vanish after several months of antiviral therapy (Hirabayashi et al. 2013). Decrease of HHV-6 DNA in the CSF over time has been documented, but some patients were left with lingering neurological compromise. Epilepsy may develop subsequent to HHV-6 encephalitis, which is refractory to multiple antiepileptic drugs (Al-Zubeidi et al. 2014; Kawamura et al. 2013; Raspall-Chaure et al. 2013). An HIV-1-infected patient with HHV-6-associated encephalitis died despite cidofovir treatment (Astriti et al. 2006). Therapeutic failure after ganciclovir (Chan et al. 2002; Imataki and Uemura 2015) or foscarnet (Holden and Vas 2007) application has been reported. Most frequently, mutation of the phosphotransferase gene results in ganciclovir resistance (Imataki and Uemura 2015). HHV-7 is much less susceptible to ganciclovir treatment than HHV-6, although full recovery from encephalitis has been described (Miranda et al. 2011). In two children with HHV-6 encephalitis, seemingly healthy but carrying genetic mitochondrial disorders, neurological symptoms did not cease following initiation of ganciclovir or foscarnet medication due to HHV-6B-associated encephalitis (Al-Zubeidi et al. 2014). Mutants resistant to foscarnet and cidofovir have already been described (Prichard and Whitley 2014). In a Japanese study of allogeneic stem cell transplant patients, low-dose foscarnet did not suppress HHV-6 reactivation sufficiently to prevent all cases of HHV-6 encephalitis (Ogata et al. 2013a). Both foscarnet and valganciclovir long-term therapy of ciHHV-6 patients with CNS disturbances have been reported to be successful resulting in a rapid fall in CSF and blood viral load and no clinical sequele (Kobayashi et al. 2011; Montoya et al. 2012; Pantry et al. 2013; Troy et al. 2008; Wittekindt et al. 2009), although no clinical trials have been conducted. HHV-6B, but not HHV-6A, was shown to be resistant to the antiviral effects of IFN-alpha and -beta due to silencing of IFN-stimulated genes (Jaworska et al. 2010). Clinical and virus free recovery was achieved in a 11-year-old immunocompetent boy with HHV-7-associated hemorrhagic brainstem encephalitis by applying IVIG, corticosteroids, and plasma exchange but no antiviral medication (Fay et al. 2015).

Prophylactic ganciclovir can prevent HHV-6 reactivation (Zerr 2006). Pre-emptive ganciclovir (Ogata et al. 2013a) or foscarnet (Ishiyama et al. 2011) therapy was shown to be successful in many, but not all, cases of HHV-6 or CMV encephalitis in transplanted patients (Ishiyama et al. 2011; Ljungman 2002). A recent study showed that high-dose valacyclovir may be helpful as a prophylaxis against HHV-6 reactivation (Hill et al. 2015a, b). So far, no consensus has been reached regarding appropiate preventive methods (Ogata et al. 2013a). The recent guidelines from the American Society of Transplantation do not recommend antiviral prophylaxis for HHV-6 infection, but for manifested disease, especially encephalitis, consideration of intravenous ganciclovir and foscarnet are recommended (Lautenschläger and Razonable 2012).

A lipophilic derivate of cidofovir, CMX001 (Brincidofovir) can inhibit replication of both HHV-6A (strain GS) and HHV-6B (strains Z29 and HST) in cell cultures (Bonnafous et al. 2013). In a phase 2 trial, it was found to prevent CMV disease in HCT recipients (Marty et al. 2013), although a subsequent phase III trial was suspended due to higher GVHD in the arm with active drug compared to the control arm (Chimerix 2015). Brincidofovir could prevent reactivation of a broad range of viruses causing complications after HSCT including adenovirus and could be used before the exact causative agent of encephalitis is verified (Prichard and Whitley 2014). The development of HHV-6 specific cytotoxic T cells for adoptive immunotherapy has been encouraging. Several phase II and phase III clinical trials with other drugs are in progress (see references in Ogata et al. 2015; references in Prichard and Whitley 2014). Antiviral drugs have serious side effects. Ganciclovir can cause serious bone marrow suppression and foscarnet can cause renal toxicity (Ward 2005; Hill et al. 2015a, b). As a curiosity, detrimental effect of ganciclovir on the residual POLG activity in two immunocompetent children carrying mutations and HHV-6B-associated encephalitis has been described (Al-Zubeidi et al. 2014). Application of drugs with less toxicity are needed for patients with severe underlying conditions (Papadopoulou et al. 2014).

## Conclusions

Roseoloviruses are important pathogens in immunocompetent young people and immunocompromised individuals, especially in the transplantation setting, and cause serious and lethal complications including CNS disorders. The scale of the contribution of Roseolovirus infection to diverse neurological diseases has not been appreciated in the past (Steiner et al. 2010), but such cases must now be fully investigated for these viruses. Individual case studies must be replaced by properly selected cohorts with standardised diagnostic procedures and adequate antiviral treatment. HHV-6B is more prevalent than HHV-6A in cases of encephalitis and encephalopathy in both immunocompetent and immunocopromised individuals. Simultaneous primary betaherpesvirus infections, and HHV-6 and/or CMV reactivation by HHV-7 have been found in several cases of encephalitis and/or encephalopathy. Hidden genetic disorders, e.g., defects of mitochodrial enzymes might predispose small children to encephalitis or encephalopathy during primary HHV-6B or HHV-7 infection. Male gender, as well as young age for HHV-6B, but advanced age for HHV-7 contribute to CNS disorders. In the transplantation setting, certain HLA classs I alleles, advanced underlying malignant diseases, but above all, UCBT, and repeated SCT/HSCT are the most important risk factors for roseolovirus-associated encephalitis. The clinical course and sequelae seem to be more severe at any age in HHV-7-associated CNS involvements than in HHV-6-associated forms. Clinical symptoms, sophisticated neuroimaging procedures and laboratory findings in the blood and CSF are very diverse and vary by individual and differ in primary or reactivated infections. Direct cell destruction due to virus infection in the limbic system, brain stem, temporal lobe combined with damages attributed to proinflammatory cytokines elicit limbic encephalitis, and several other forms of brain damages. For differential diagnostic purposes, mPCR is recommended, while qPCR is used to monitor blood and CNS copy number of the actual pathogen(s) during disease course and to predict efficacy of treatment and outcome (Gautheret-Dejean et al. 2013). Both exogenous and chromosomally integrated HHV-6A and HHV-6B have to be distinguished by molecular methods. CiHHV-6 has been detected in immunocompetent persons with both mild and severe CNS disorders. Due to a lack of controlled trials, empirical therapy of roseolovirus-associated encephalitis has been successful when started very early in the disease course or in a preemptive approach. Especially in ciHHV-6 patients, long-term treatment is required (Pantry et al. 2013). Ganciclovir and foscarnet are recommended as first-line agents, and cidofovir is in the second line of defense because its ability to penetrate the CNS has been poorly studied. Frequent delays due to late diagnosis, inadequate (e.g., acyclovir), or no medication, unavoidable treatment of serious underlying diseases with drug combinations—that have roseolovirus-activating potential—emergence of resistant betaherpesvirus mutants in single or simultaneous infections (Agut 2011) or the presence of co-pathogens (Steiner et al. 2010) maintain high mortality rates. New drugs with wide antiviral activity (e.g., brincidofovir) and their combination with immunotherapy (e.g., IVIG) or other types of therapy are required to combat roseolovirus-associated CNS complications on a very carefully balanced, individual basis.
